# Hepatic Hypoxia-Inducible Factor 1α Mediates Ferroptosis via Transferrin Receptor 1 in Acute Liver Injury

**DOI:** 10.3390/antiox15010081

**Published:** 2026-01-08

**Authors:** Jiayu Yang, Meicheng Wang, Shichao Cui, Yulan Xia, Yinfang Xie, Zhu Hu, Ni Li, Xinwen Zhang, Pengfei Zhu, Xu Yu, Linshi Wu, Jingya Li

**Affiliations:** 1 School of Pharmaceutical Science and Technology, Hangzhou Institute for Advanced Study, University of Chinese Academy of Sciences, Hangzhou 310024, China; yangjiayu21@mails.ucas.ac.cn (J.Y.); xieyinfang23@mails.ucas.ac.cn (Y.X.); 2 University of Chinese Academy of Sciences, Beijing 100049, China; s20-wangmeicheng@simm.ac.cn (M.W.); xiayulan@simm.ac.cn (Y.X.); 3 State Key Laboratory of Drug Research, The National Center for Drug Screening, Shanghai Institute of Materia Medica, Chinese Academy of Sciences, 189 Guo Shou Jing Road, Shanghai 201203, China; sccui@simm.ac.cn (S.C.); lini@simm.ac.cn (N.L.); xwzhang@simm.ac.cn (X.Z.); 4 State Key Laboratory of Drug Research, Ethnomedicine and Biofunctional Molecule Research Center, Shanghai Institute of Materia Medica, Chinese Academy of Sciences, Shanghai 201203, China; huzhu@simm.ac.cn; 5 Department of Endocrinology, Affiliated Hospital of Nanjing University of Chinese Medicine, Nanjing 210029, China; pfzhu34@foxmail.com (P.Z.); yuxu2017@126.com (X.Y.); 6 Department of Biliary-Pancreatic Surgery, Renji Hospital, School of Medicine, Shanghai Jiao Tong University, Shanghai 200127, China

**Keywords:** hypoxia-inducible factor-1α, acute liver injury, ferroptosis, transferrin receptor 1

## Abstract

Acute liver injury (ALI) is a potentially life-threatening condition lacking effective clinical drugs. Hypoxia-inducible factor-1α (HIF-1α) is a key regulator of both inflammation and metabolism. In ALI, HIF-1α expressions are upregulated, but the role of HIF-1α in hepatocytes and whether it can be targeted remain unclear. Herein, clinical samples and ALI murine models including lipopolysaccharide/D-galactosamine (LPS/D-GalN), acetaminophen (APAP), and thioacetamide (TAA) revealed an increase in HIF-1α expression and ferroptosis. Using HIF-1α gain and loss of function mouse and hepatocyte culture models, we demonstrated that HIF-1α upregulation exacerbated liver ferroptosis and injury. Mechanistically, HIF-1α/transferrin receptor protein 1 (TFR1) axis drives hepatic iron overload, promoting ferroptotic cell death and liver injury. In addition, TFR1 inhibition reversed HIF-1α-induced ALI. Importantly, pharmacological inhibition of HIF-1α and TFR1 significantly reduced ferroptosis and mitigated liver injury both in vivo and in vitro. Together, our findings demonstrate the pathological role of hepatic HIF-1α, which may serve as a promising target of therapeutic intervention.

## 1. Introduction

Acute liver injury (ALI) is a potentially life-threatening condition characterized by rapid progression and massive hepatocyte death, with limited treatment options available [1]. It can present in a background of healthy and normal liver or as acute-on-chronic liver failure (ACLF) in the presence of any chronic liver disease or cirrhosis [2,3]. The etiology of ALI varies according to the socioeconomic status of the country. In developing countries, ALI is caused mainly by viral hepatitis (hepatitis A-E viruses), whereas drugs such as acetaminophen (APAP) are the most common cause of ALI in developed countries [4]. Despite the variable etiology and severe condition of ALI, current therapies are limited. Hence, studies aimed at exploring therapeutic targets and underlying mechanisms are urgently needed [4,5].

Several crucial factors have been described in the pathogenesis of ALI including hypoxia, cell death, oxidative stress, and inflammation [6,7,8]. During hypoxia, hypoxia-inducible factor 1α (HIF-1α) plays a key role to regulate a wide range of genes involved in energy metabolism, cell survival, angiogenesis, iron homeostasis, and inflammation [9,10,11]. A previous study has shown that HIF-1α inhibition and myeloid-specific HIF-1α deletion protect against inflammation-induced ALI [5,12], suggesting a pathological role of HIF-1α in myeloid-specific cells and inflammation in ALI. In addition, HIF-1α knockout significantly alleviates APAP-induced liver injury in the presence of inflammation and oxidative stress [13], indicating a negative regulation of HIF-1α in hepatic injury during ALI. Due to the therapeutic potential of HIF-1α targeting, it is crucial to fully illustrate the pathological role of HIF-1α based on cell-type and pathology specificity.

Ferroptosis is a distinct form of programmed cell death, which is relative to ALI condition according to recent reports [14]. Besides lipid peroxidation, recent studies have revealed that intracellular iron level and metabolism play important roles in ferroptosis [15]. While hepatocytes are crucial for iron homeostasis, hepatic dysregulation of iron metabolism could exacerbate liver injury. Excess iron is hazardous by promoting lipid peroxidation and oxidative damage to intracellular molecules (lipid, DNA, or protein) through the Fenton reaction, which is observed in patients with liver failure [16,17,18]. Notably, transferrin receptor protein 1 (TFR1) is essential for cellular iron uptake; it binds to iron carrier transferrin, contributing to the cellular iron pool through the endosomal cycle and thus playing a key role in ferroptosis [19]. In ALI, TFR1 expression is markedly upregulated in the liver [17,20,21,22], and the inhibition or knockdown of TFR1 significantly alleviates ferroptosis in hepatocytes [17]. Given the critical role of TFR1 in ferroptosis and ALI, its upstream regulatory mechanisms warrant detailed elucidation.

Previous studies suggested that HIF-1α may transcriptionally regulate the TFR1 gene, implying the significance of the HIF-1α/TFR1 signaling pathway in ferroptosis and ALI [23,24]. However, studies show that the effect of HIF-1α on ferroptosis is based on tissue and cell specificity. HIF-1α could induce ferroptosis by activating HO-1, leading to damage in hippocampal neurons, testes, and renal tubules [23,25,26,27], while other studies have shown that HIF-1α suppresses ferroptosis through regulating SLC1A1 or fatty acid binding proteins [28,29,30]. Whether HIF-1α regulates hepatic ferroptosis in ALI conditions and the underlying mechanisms remain unclear.

Here, we explored the role of HIF-1α in hepatocytes in ALI and in the livers of patients with ACLF. Our study showed that the overexpression of hepatocyte HIF-1α (HepHIF-1α^LSL/LSL^) significantly aggravated lipopolysaccharide/D-galactosamine (LPS/D-GalN)-induced ALI, whereas the hepatocyte-specific knockout of HIF-1α (HIF-1α KO^Hep^) markedly alleviated ALI. In addition, we determined that hepatic HIF-1α regulated iron uptake via TFR1 to mediate hepatocyte ferroptosis in ALI, which is similarly upregulated in drug and toxin-induced ALI condition. Notably, pharmacological inhibition of both HIF-1α and TFR1 significantly attenuated liver ferroptosis and ALI. Taken together, these findings identify HIF-1α/TFR1 as a key regulation axis of ferroptosis in hepatocytes under ALI condition and provide a potential therapeutic target for pharmaceutical agents in ALI.

## 2. Materials and Methods

Additional details for all methods and reagents are provided in the Supplementary Information.

### 2.1. Human Liver Tissue Collection

Paraffin-embedded human liver sections from Healthy Controls and ACLF patients were provided by the histology department at Renji Hospital, Shanghai Jiao Tong University, Shanghai, China. The Renji Hospital Ethics Committee at Shanghai Jiao Tong University School approved the collection of human samples during hepatic resection or liver transplantation and the experiments conducted in this study (approval numbers: (2014)148 k and (2016)142 k).

The transplanted livers were voluntarily donated and allocated by the China Organ Transplant Response System or sourced from living-related party liver transplantation approved by the ethics committee. Written informed consent was acquired from donors or their legal surrogates.

### 2.2. Animal Model and Approval

All animal studies had the approval of the Institutional Animal Care and Use Committee of the Shanghai Institute of Materia Medica, Chinese Academy of Science.

C57BL/6J mice (male, 7–8 weeks old) were purchased from Beijing HFK Bio-Technology Co., Ltd. (ChangPing District, Beijing, China). Hepatocyte-specific HIF-1α knockout (HIF-1α-KO^hep^) mice and HepHIF-1α^LSL/LSL^ mice (C57BL/6J background) were generated, as described in the Supplementary Information. Male mice aged 8–10 weeks were used to induce ALI according to published methods [1,5]. Briefly, the mice were intraperitoneally injected with 100 μg/kg body weight LPS (L2630, Sigma, Saint Louis, MI, USA) and 700 mg/kg body weight D-GalN (G0500, Sigma, Saint Louis, MI, USA) for indicated time.

ALI was induced by APAP in C57BL/6J mice (male, 8–10 weeks old) according to published research [13]. The mice were starved for 12 h and then intraperitoneally injected with 300 mg/kg APAP (Abs44055999, Absin, Shanghai, China) for 6 h.

ALI was induced by TAA in mice (male, 8–10 weeks old) according to published research [31]. The mice were intraperitoneally (i.p.) injected with 100 mg/kg TAA (Abs42028342, Absin, Shanghai, China) for 24 h.

Detailed information is available in the Supplementary Information.

### 2.3. Liver Injury and Histological Analyses

Plasma lactate dehydrogenase (LDH), alanine aminotransferase (ALT), and aspartate aminotransferase (AST) levels were measured by a JCA-BM6010/C Automatic Analyzer (JEOL, Tokyo, Japan) according to the manufacturer’s instructions.

Histological pathology was performed as a standard protocol [32]. Briefly, liver tissues were collected and routinely embedded into paraffin. Liver sections were stained with hematoxylin staining (Ribiology, Shanghai, China; Yangming Medical Laboratory, Ningbo, China). The iron distribution in tissues was measured by 3,3′-diaminobenzidine (DAB)-enhanced Perls’ staining [22,33] (Yangming Medical Laboratory, Ningbo, China). The histological features of the tissues were observed under Brightfield and Fluorescence Slide Scanning System (Shenzhen Shengqiang Technology Co., Ltd., Shenzhen, China) and imaged.

### 2.4. Isolation and Culture of Primary Hepatocytes

Primary hepatocytes were isolated from male C57BL/6J mice at 6–8 weeks of age. The mice were anesthetized and perfused with perfusion buffer and collagenase-I (0.48 mg/mL, LS004196, Worthington, Lakewood, NJ, USA) through the portal vein at 37 °C. The liver of each mouse was cut, dispersed, filtered through a 70 mm cell strainer (Thermo Fisher Scientific, Waltham, MA, USA), and spun at 700 r/min for 5 min at 4 °C. The cells were then resuspended in a Hepato ZYME-SFM (17705021, GIBCO, Grand Island, NY, USA) medium and plated at the indicated density in a culture plate [32].

Primary hepatocytes were seeded in 96-well plates at the density of 2 × 10^4^ for 6 h in a mixture of low-glucose and adherent culture medium (4:1), supplemented with 10% FBS, 1× P/S, and 1 × Glutathione. Primary hepatocytes were then cultured overnight in William’s E medium containing 5% FBS, 1 × P/S, and 10 mM HEPES prior to any treatments [18].

### 2.5. RNA Sequencing

TRIzol(9109, Takara, Shiga, Japan) was used to isolate RNA from the liver. RNA sequencing and bioinformatics analysis were performed by APExBIO Technology LLC (Houston, TX, USA).

### 2.6. Statistical Analysis

Statistical analyses were performed using GraphPad Prism 8.0, and data are presented as mean ± SEM. Analytical details are provided in the Supplementary Information.

## 3. Results

### 3.1. Increased Hepatic HIF-1α Expression Is Correlated with Ferroptosis in ALI

**Figure 1:**
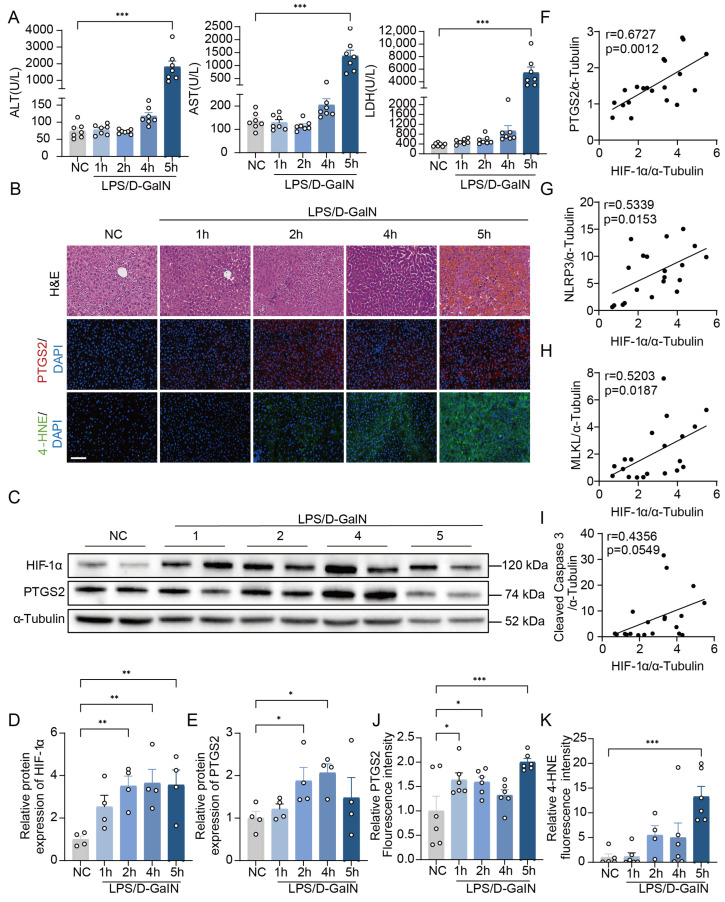
**Increased HIF-1α expression is correlated with ferroptosis in the livers of mice with LPS/D-GalN-induced ALI.** (**A**) Plasma ALT, AST, and LDH at different timepoints post LPS/D-GalN injection (*n* = 7 per group). (**B**) Representative images of H&E, PTGS2, and 4-HNE staining of liver sections (*n* = 4–6 per group). Scale bar: 100 μm. (**C**–**E**) The protein levels of HIF-1α and PTGS2 in the liver of mice at different timepoints after LPS/D-GalN were assessed via Western blotting (*n* = 4 per group). (**F**–**I**) Correlations between protein levels of HIF-1α and PTGS2, NLRP3, MLKL, and Cleaved Caspase 3. (**J**,**K**) The relative fluorescence intensity of PTGS2 and 4-HNE was measured using ImageJ 1.50i (*n* = 4–6 per group). Data are displayed as means  ±  SEMs. * *p* < 0.05, ** *p* < 0.01, *** *p* < 0.001.

To evaluate the role of hepatic HIF-1α in ALI, the changes in HIF-1α expression was examined at different timepoints following LPS/D-GalN treatment. Plasma alanine aminotransferase (ALT), aspartate aminotransferase (AST), and lactate dehydrogenase (LDH) were markedly increased at 4 h post LPS/D-GalN (Figure 1A). Liver injury was observed in livers of the mice injected with LPS/D-GalN, as shown by H&E staining (Figure 1B). In addition, LPS/D-GalN-treated mice livers exhibited extensive infiltration of inflammatory cells, as shown by MPO staining and hepatic inflammatory gene expression (Supplementary Information Figure S1A–F). These results indicate that we successfully established an LPS/D-GalN-induced ALI model. Notably, HIF-1α protein levels were significantly increased at different times after the injection of LPS/D-GalN (Figure 1C,D).

Given that cell death has been found to play important roles in ALI [34], we assessed several key biomarkers for ferroptosis (prostaglandin-endoperoxide synthase 2 [35], PTGS2), apoptosis (cleaved caspase 3 [36]), pyroptosis (NOD-like receptor thermal protein domain-associated protein 3, NLRP3 [37]), and necrosis (mixed lineage kinase domain-like protein, MLKL [36]). Ferroptosis and pyroptosis occurred in the early phase (1–2 h), and apoptosis and necrosis occurred in the progressive phase (~4 h) (Figure 1C,E; Supplementary Information Figure S1G–J).

Moreover, we detected correlations between HIF-1α protein levels and ferroptosis marker (PTGS2, r = 0.6727, *p* < 0.05), pyroptosis marker (NLRP3, r = 0.5339, *p* < 0.05), and necrosis marker (MLKL, r = 0.5203, *p* < 0.05), but not with apoptosis marker (cleaved caspase 3, r = 0.4356, *p* > 0.05) (Figure 1F–I). In addition, immunofluorescence staining revealed that the levels of 4-hydroxynonenal (4-HNE, a lipid peroxidation marker) and prostaglandin G/H synthase 2 (PTGS2, a ferroptosis marker) were significantly increased in LPS/D-GalN-treated mice (Figure 1B,J,K). Thus, our data demonstrate that HIF-1α might contribute to hepatocyte ferroptosis in LPS/D-GalN-induced ALI.

### 3.2. Hepatocyte-Specific Stable HIF-1α Overexpression Exaggerates Ferroptosis and LPS/D-GalN-Induced ALI

**Figure 2:**
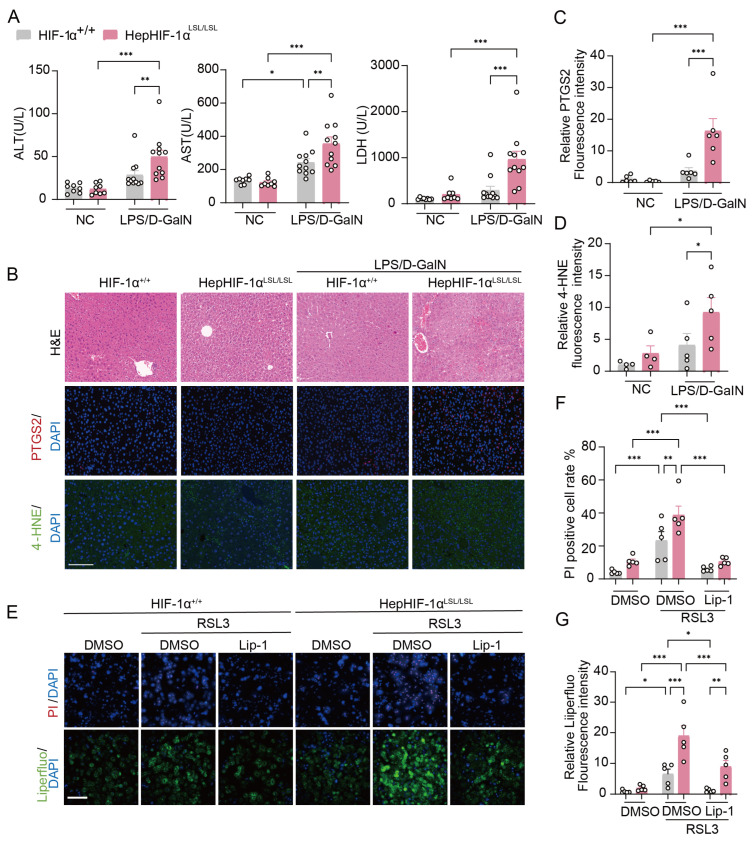
**Hepatocyte-specific HIF-1α overexpression exaggerates ferroptosis in vivo and in vitro.** (**A**) Plasma ALT, AST, and LDH at 4 h post LPS/D-GalN injection in HIF-1α^+/+^ mice and HepHIF-1α^LSL/LSL^ mice (*n* = 8 or 11 per group). (**B**) Representative images of H&E, PTGS2, and 4-HNE staining of liver sections. Scale bar: 100 μm. (**C**,**D**) Quantitative analysis of PTGS2 and 4-HNE (*n* = 4–6 per group). (**E**) Hepatocytes were isolated from HIF-1α^+/+^ and HepHIF-1α^LSL/LSL^ mice and treated with RSL3 (1 μM) or RSL3 (1 μM) + Lip-1 (2 μM) for 12 h. Representative fluorescence images of dead cells (red, PI) and lipid peroxidation (green, Liperfluo). Scale bar: 200 μm. (**F**,**G**) Quantitative analysis of PI-positive cells and Liperfluo (*n* = 5 per group). Data are displayed as means  ±  SEMs. * *p* < 0.05, ** *p* < 0.01, *** *p* < 0.001.

To explore the correlation between hepatocytes HIF-1α and ferroptosis in ALI, we used HepHIF-1α^LSL/LSL^ mice, in which the HIF-1α is stabilized in hepatocytes, leading to higher HIF-1α levels than in HIF-1α^+/+^ mice (Supplemental Figure S2A). In the mice aged 7–8 weeks, HIF-1α overexpression in hepatocytes did not affect liver function, histology, or hepatic ferroptosis (Figure 2A–D). Under LPS/D-GalN treatment, ALT and AST activities, as well as LDH levels, were significantly greater in HepHIF-1α^LSL/LSL^ mice than in HIF-1α^+/+^ mice at 4 h (Figure 2A). In addition, HepHIF-1α^LSL/LSL^ mice also presented liver histology injury, whereas levels of hepatic inflammatory genes were not affected (Figure 2B, Supplementary Information Figure S2B–E). More importantly, we found that hepatic lipid peroxidation (4-HNE) and ferroptosis marker (PTGS2) levels were significantly greater in HepHIF-1α^LSL/LSL^ mice than in HIF-1α^+/+^ mice post LPS/D-GalN injection (Figure 2B–D, Supplementary Information Figure S2F,G).

To explore the role of HIF-1α in hepatocyte ferroptosis, (1S, 3R)-RSL3 (RSL3) was utilized to simulate ferroptosis in primary hepatocytes. The results revealed that HIF-1α aggravated RSL3-induced hepatocyte death, reactive oxygen species (ROS), and lipid peroxidation (Figure 2E–G, Supplementary Information Figure S2H,I), which were markedly blocked by a potent ferroptosis inhibitor liproxstatin-1 (Lip-1), suggesting that the overexpression of hepatocyte HIF-1α exacerbated RSL3-induced cell death via ferroptosis. These results indicate that the overexpression of hepatocyte HIF-1α may aggravate LPS/D-GalN-induced ALI by mediating ferroptosis in vitro and in vivo.

### 3.3. Hepatic HIF-1α Regulates TFR1 Expression and Iron Homeostasis in LPS/D-GalN-Induced ALI

**Figure 3:**
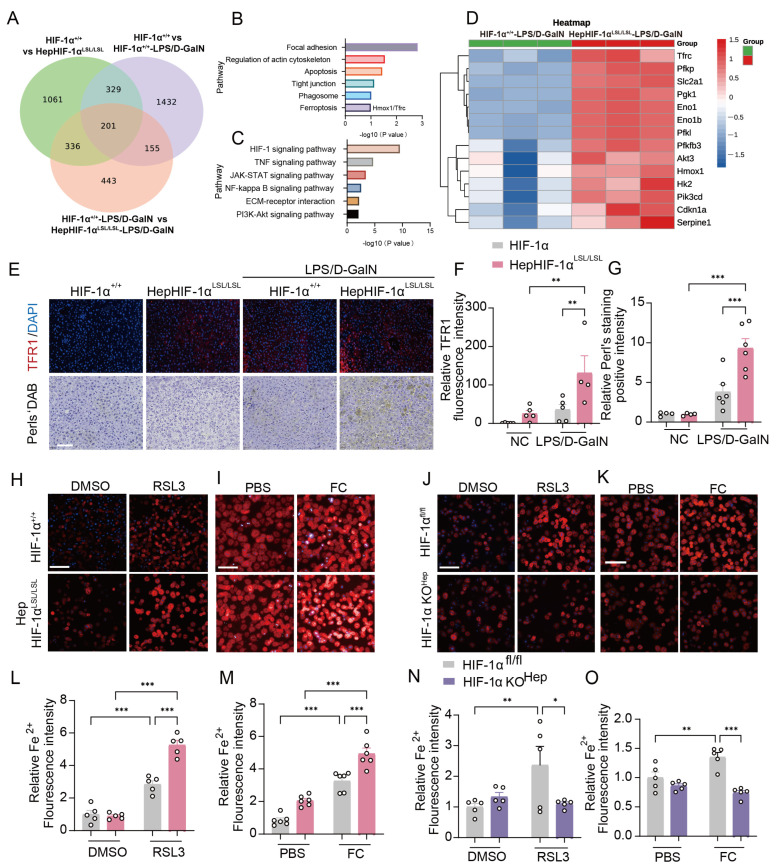
**Hepatic HIF-1α regulates TFR1 expression and iron metabolism in ALI.** (**A**) Venn diagrams showing overlap among the results obtained for the 3 groups (HIF-1α^+/+^ vs. HepHIF-1α^LSL/LSL^, HIF-1α^+/+^ vs. HIF-1α^+/+^-LPS/D-GalN, and HIF-1α^+/+^-LPS/D-GalN vs. HepHIF-1α^LSL/LSL^-LPS/D-GalN). (**B**,**C**) KEGG analysis of 201 overlapping differentially expressed genes. (**D**) Heatmaps showing ferroptosis-related genes among the upregulated HIF-1α-targeted genes. (**E**) Representative images of TFR1 and Perls’ DAB staining of liver sections at 4 h after LPS/D-GalN injection in HIF-1α^+/+^ and HepHIF-1α^LSL/LSL^ mice. Scale bar: 100 μm. (**F**,**G**) Quantitative analysis of TFR1 and Perls’ DAB staining (*n* = 4–6 per group). (**H**,**L**) Representative fluorescence images of FerroOrange staining of hepatocytes from HIF-1α^+/+^ and HepHIF-1α^LSL/LSL^ mice treated with RSL3 (1 μM) for 12 h (**H**) and quantitative analysis of FerroOrange (**L**). (**I**,**M**) Representative fluorescence images of FerroOrange staining of hepatocytes from HIF-1α^+/+^ and HepHIF-1α^LSL/LSL^ mice treated with FC (100 μM) for 12 h (**I**) and quantitative analysis of FerroOrange (**M**). (**J**,**N**) Representative fluorescence images of FerroOrange staining of hepatocytes from HIF-1α^fl/fl^ and HIF-1α KO^Hep^ mice treated with RSL3 (1 μM) for 12 h (**J**) and quantitative analysis of FerroOrange (**N**). (**K**,**O**) Representative fluorescence images of FerroOrange staining of hepatocytes from HIF-1α^fl/fl^ and HIF-1α KO^Hep^ mice treated with FC (100 μM) for 12 h (**K**) and quantitative analysis of FerroOrange (**O**). *n* = 5–6 per group (**H**–**K**). Data are displayed as means  ±  SEMs. * *p* < 0.05, ** *p* < 0.01, *** *p* < 0.001.

As a transcriptional activator, HIF-1α can bind the enhancer of the target genes to promote transcription, resulting in target gene expression [38]. To elucidate the mechanism by which HIF-1α mediates ferroptosis, we conducted RNA sequencing of the liver to analyze whether target gene expression promoted by HIF-1α regulates ferroptosis in hepatocytes. Differential gene expression (DEG) analysis between HepHIF-1α^LSL/LSL^ and HIF-1α^+/+^ mice in both LPS/D-GalN group and control groups revealed 201 overlapping different expression genes (Figure 3A). Kyoto Encyclopedia of Genes and Genomes (KEGG) enrichment analysis was performed using the 201 overlapping genes, and the results revealed that several pathways and processes including focal adhesion, apoptosis, ferroptosis, and the HIF-1 signaling pathway, were affected (Figure 3B,C; Supplementary Information Figure S3A–C). In addition, the ferroptosis-related genes, *Tfrc* and *Hmox1*, were among the upregulated HIF-1α-targeted genes (Figure 3B,D); thus, the phenotype of exaggerated ferroptosis in hepHIF-1α^LSL/LSL^ mice may be explained by the altered regulation of *Tfrc* or *Hmox1*. We therefore examined whether the TFR1 or HO-1 protein plays a role in HepHIF-1α^LSL/LSL^ mice after LPS/D-GalN injection. Compared with those in HIF-1α^+/+^ mice, TFR1 protein expression was significantly increased in HepHIF-1α^LSL/LSL^ mice (Figure 3E,F; Supplementary Information Figure S3D,E). Given that TFR1 plays a critical role in iron homeostasis and ferroptosis [17], we examined iron levels in the liver via 3,3′-diaminobenzidine (DAB)-enhanced Perls’ staining of liver sections [22]. More iron accumulation was detected in the liver of HepHIF-1α^LSL/LSL^ mice (Figure 3E,G). Furthermore, TFR1 expression and hepatic iron levels were measured at different timepoints following LPS/D-GalN treatment. The results revealed that TFR1 accumulation occurred in the early phase (1–2 h), and more TFR1 accumulation and iron overload occurred in the progressive phase (Supplementary Information Figure S3G–J), which was consistent with the protein expression of HIF-1α.

In addition, to investigate whether HIF-1α promotes ferroptosis by regulating iron homeostasis via TFR1, the Fe (II)-selective fluorescent probe FerroOrange [39,40] was employed to measure intracellular Fe (II) during RSL3-induced ferroptosis in primary hepatocytes. Consistent with the in vivo results, we observed a greater level of ferrous ions after RSL3 treatment in HepHIF-1α^LSL/LSL^ hepatocytes (Figure 3H,L), and a lower level of ferrous ions and ferroptosis in HIF-1α KO^Hep^ hepatocytes (Figure 3J,N; Supplementary Information Figure S4A–D). To investigate the role of HIF-1α in iron homeostasis during ferroptosis, we treated primary hepatocytes with 100 μM ferric citrate (FC) for 12 h [15,18,41,42,43]. The data revealed that FC-induced cell death, ROS, lipid peroxidation, and ferrous iron accumulation in primary hepatocytes could be aggravated by HIF-1α overexpression (Figure 3I,M, Supplementary Information Figure S5A–D) and alleviated by HIF-1α knockout (Figure 3K,O, Supplementary Information Figure S4E–H). These findings underscore the essential function of the HIF-1α/TFR1 axis in regulating iron metabolism and ferroptosis in the liver.

### 3.4. Hepatocyte-Specific HIF-1α Knockout Mitigates Ferroptosis and LPS/D-GalN-Induced ALI via TFR1

We next performed in vitro experiments to verify the role of TFR1 in HIF-1α-induced ferroptosis. The small molecular compound Ferristatin II (Fer II) has been reported to regulate iron homeostasis through the degradation of TFR1 [41,44]. We found that Fer II reversed the increase in ferrous iron accumulation, ROS, lipid peroxidation, and ferroptosis in HepHIF-1α^LSL/LSL^ hepatocytes in response to FC- and RSL3-induced ferroptosis (Supplementary Information Figures S6A–E and S7A–E). In mice with LPS/D-GalN injection, treatment with Fer II markedly improved liver function (AST, ALT, and LDH) and decreased liver injury and levels of hepatic inflammatory genes (Supplementary Information Figure S8A–F). More importantly, hepatic TFR1 mRNA and protein levels, ferrous iron accumulation, lipid peroxidation levels (4-HNE), and the expression of the ferroptosis marker (PTGS2) in the liver decreased after Fer II treatment (Supplementary Information Figure S8B,G–M). These results suggest a critical role of the HIF-1α/TFR1 axis in hepatocyte ferroptosis.

**Figure 4:**
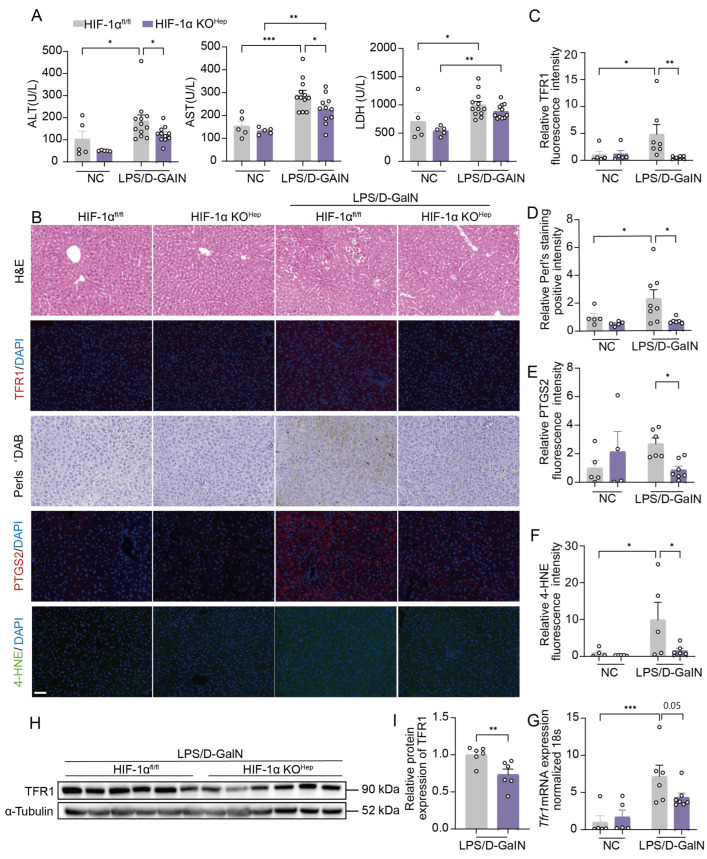
**Hepatocyte-specific HIF-1α ablation mitigates ferroptosis and LPS/D-GalN-induced ALI**. (**A**) Plasma ALT, AST, and LDH at 4 h post LPS/D-GalN injection in HIF-1α^fl/fl^ mice and HIF-1α KO^Hep^ mice (*n* = 5–12 per group). (**B**) Representative images of H&E, TFR1, Perls’ DAB, PTGS2, and 4-HNE staining of liver sections at 4 h after LPS/D-GalN injection in HIF-1α^fl/fl^ mice and HIF-1α KO^Hep^ mice. Scale bar: 100 μm. (**C**–**F**) Quantitative analysis of TFR1, Perls’ DAB, PTGS2, and 4-HNE (*n* = 4–8 per group). (**G**) *Tfr1* mRNA expression in the liver was determined by qPCR (*n* = 5–7 per group). (**H**) Representative Western blot analysis of the hepatic expression levels of TFR1 (*n* = 6 per group). (**I**) The relative protein level of TFR1 was measured using ImageJ 1.50i (*n* = 6 per group). Data are displayed as means  ±  SEMs. * *p* < 0.05, ** *p *< 0.01, *** *p* < 0.001.

To clarify the role of HIF-1α in hepatocyte, we generated HIF-1α KO^Hep^ mice. As shown by the qPCR analysis results, the mRNA level of HIF-1α significantly reduced in the liver of HIF-1α KO^Hep^ mice (Supplementary Information Figure S9A), and the HIF-1α KO^Hep^ did not affect liver function, histology, or hepatic ferroptosis (Figure 4A–F). In contrast to HepHIF-1α^LSL/LSL^ mice, HIF-1α KO^Hep^ mice presented significant alleviation of liver injury, including alleviated liver function (AST, ALT, and LDH) and decreased levels of hepatic inflammatory genes (Figure 4A, Supplementary Information Figure S9E–H). In addition, the hepatocyte-specific knockout of HIF-1α markedly decreased hepatic lipid peroxidation (4-HNE) and ferroptosis marker (PTGS2) levels of LPS/D-GalN-injected mice (Figure 4B,E,F, Supplementary Information Figure S9B–D). Moreover, hepatic TFR1 mRNA and protein levels, as well as ferrous iron accumulation induced by LPS/D-galN were decreased in HIF-1α KO^Hep^ mice (Figure 4B–D,G–I). Furthermore, HIF-1α knockout decreased ferroptosis, including lipid peroxidation and ROS in primary hepatocytes treated with RSL3 and FC in vitro (Figure 3J,N,K,O; Supplementary Information Figure S4). Collectively, these findings indicate that hepatocyte HIF-1α mediates ferroptosis and liver injury in ALI via TFR1.

### 3.5. Pharmacological Inhibition of Hepatic HIF-1α Expression Attenuates Ferroptosis in ALI

**Figure 5:**
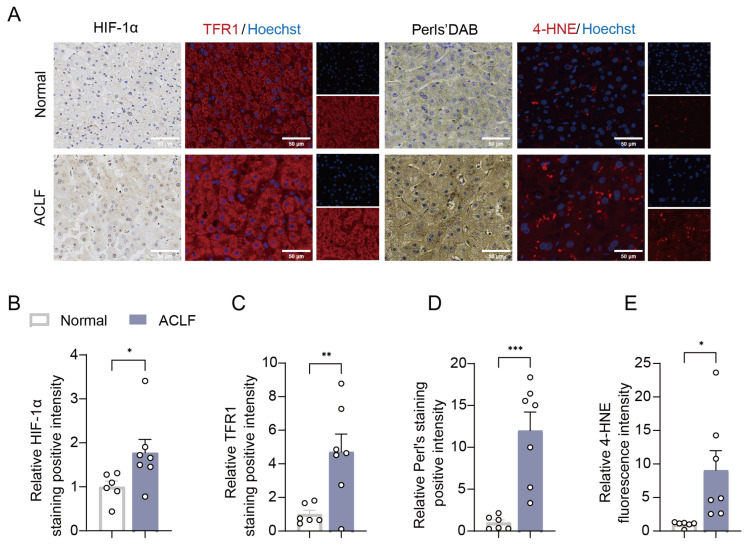
**Hepatic HIF-1α/TFR1 axis activation in patients with liver injury.** (**A**) Representative images of HIF-1α, TFR1, Perls’ DAB, and 4-HNE staining of liver sections from normal and ACLF patients. Scale bar represents 20 μm. (**B**–**E**) Quantitative analysis of HIF-1α, TFR1, Perls’ DAB, and 4-HNE staining (*n* = 6–7 per group). Data are displayed as means  ±  SEMs. * *p* < 0.05, ** *p* < 0.01, *** *p* < 0.001.

To clarify the role of hepatic HIF-1α/TFR1 pathway in ALI, we examined its expression in liver specimens from clinical patients. Thirteen liver samples from patients who underwent hepatic resection or liver transplantation were retrospectively studied with immunohistology. Among these, seven samples were diagnosed with acute-on-chronic liver failure (ACLF), while the remaining six were used as healthy control without ACLF. The patients’ information is provided in Supplementary Information (Table S4). Our results revealed that HIF-1α and TFR1 expressions were markedly upregulated in liver samples of ACLF patients (Figure 5A–C). Similarly, ferrous iron accumulation and lipid peroxidation were increased in ACLF livers (Figure 5A,D,E). This clinical evidence suggests that the activation of the hepatic HIF-1α/TFR1 axis may contribute to the development of ALI.

**Figure 6:**
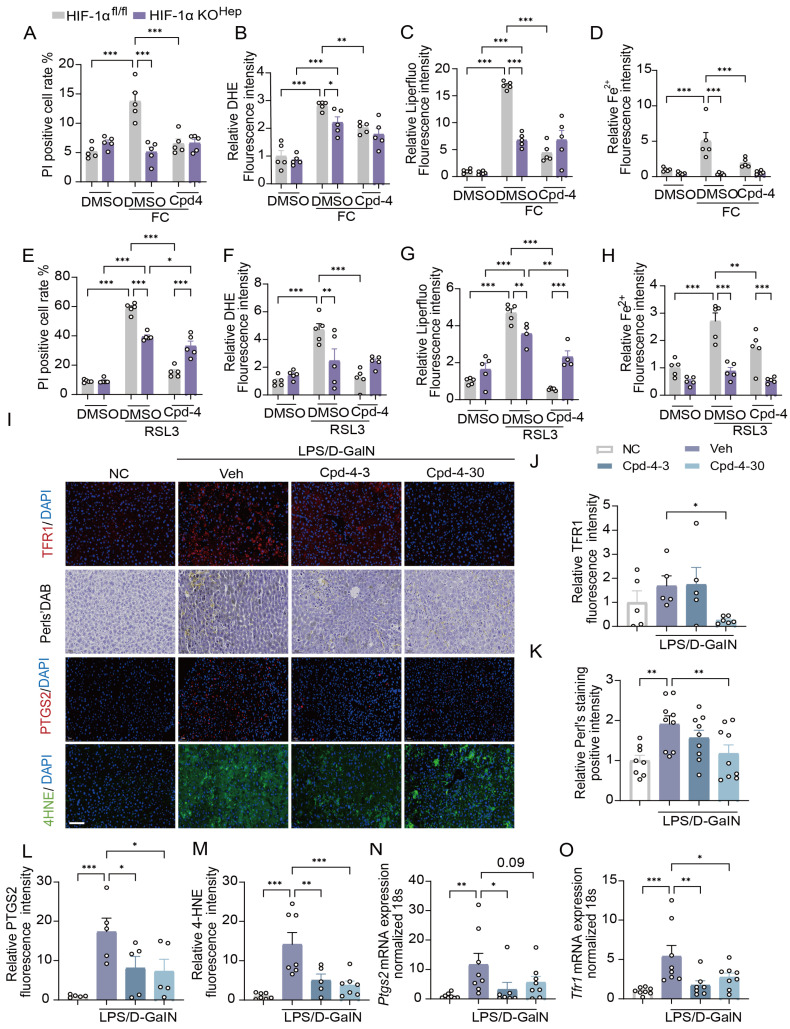
**The HIF-1α inhibitor Cpd-4 alleviates hepatocyte ferroptosis in an HIF-1α-dependent manner.** (**A**–**D**) Hepatocytes were isolated from HIF-1α^fl/fl^ mice and HIF-1α KO^Hep^ mice pretreated with Cpd-4 (10 μM) for 12 h and then treated with FC or FC + Cpd-4 for 12 h. Quantitative analysis of PI-positive cells, DHE, Liperfluo, and FerroOrange (*n* = 5 per group). (**E**–**H**) Hepatocytes were isolated from HIF-1α ^fl/fl^ mice and HIF-1α KO ^Hep^ mice pretreated with Cpd-4 (10 μM) for 12 h and then treated with RSL3 (1 μM) or RSL3+ Cpd-4 for 12 h. Quantitative analysis of PI-positive cells, DHE, Liperfluo, and FerroOrange (*n* = 4–5 per group). (**I**) Mice were injected with LPS/D-GalN after the administration of Cpd-4 (3, 30 mg/kg, po) three times. Representative images of TFR1, Perls’ DAB, PTGS2, and 4-HNE staining of liver sections. Scale bar: 100 μm. (**J**–**M**) Quantitative analysis of TFR1, Perls’ DAB, PTGS2, and 4-HNE staining (*n* = 5–6 per group for J; *n* = 8–9 per group for K; *n* = 5 per group for L; *n* = 5–7 per group for M). (**N**,**O**) The mRNA expression of *Ptgs2* and *Tfr1* were determined by qPCR (*n* = 7–8 per group). Data are displayed as means  ±  SEMs. * *p* < 0.05, ** *p* < 0.01, *** *p* < 0.001.

Our previous study found a new class of potent HIF-1α inhibitor (Cpd-4), which was isolated and characterized from a Chinese medicinal plant *P. franchetianus* and can protect ALI via suppressing the production of IL-1β in macrophage [45].To demonstrate that inhibiting the HIF-1α/TFR1 axis can protect against hepatocyte ferroptosis and alleviate the symptoms of ALI, compound-4(Cpd-4) was used ex vivo and in vivo. Cpd-4 significantly decreased cell death, ROS, lipid peroxidation, and ferrous iron accumulation in primary hepatocytes from HIF-1α^fl/fl^ mice treated with FC or RSL3, but not from HIF-1α KO^Hep^ mice (Figure 6A–H, Supplementary Information Figure S10A,B). These results suggest that Cpd-4 attenuates hepatocyte ferroptosis in an HIF-1α-dependent manner.

To assess whether Cpd-4 improves acute liver injury via hepatic ferroptosis, we administered Cpd-4 (3 or 30 mg/ kg, p.o., once per day) in LPS/D-GalN-induced ALI. As expected, Cpd-4 treatment obviously attenuated ALI, as shown by improved liver function (AST, ALT, and LDH) and liver histology (H&E) (Supplementary Information Figure S11A,B). Notably, hepatic TFR1 protein and mRNA levels, ferrous iron accumulation, lipid peroxidation (4-HNE), and ferroptosis marker (PTGS2) levels in the liver decreased after Cpd-4 treatment (Figure 6I–O, Supplementary Information Figure S11C,D). In addition, treatment with Cpd-4 decreased the expression of hepatic proinflammatory genes (Supplementary Information Figure S11E–H).

Thus, in vivo and in vitro evaluations of Cpd-4 revealed that the inhibition of HIF-1α effectively improved hepatocyte ferroptosis and ALI, suggesting a potential therapeutic target for ALI based on the protection of hepatocyte ferroptosis.

### 3.6. Hepatic HIF-1α/TFR1 Axis Activation in APAP and TAA -Induced Experimental ALI Models

The causes of ALF, such as viruses, drugs, toxins, and others, determine the specific treatment and prognosis for each patient [8]. Studies have been reported that in animal models induced by APAP (drug) and TAA (toxin), which are the most commonly used preclinical models for studying ALI, ferroptosis of hepatocytes appears to fasten disease progression [17,20,21,22,46,47]. In order to explore whether the hepatic HIF-1α/TFR1 axis activation contributes to ALI of different etiology, APAP and TAA were used to induce ALI (Supplementary Information Figures S12A,B and S13A,B). Surprisingly, the protein expressions of HIF-1α and TFR1 were markedly increased in livers of mice injected with APAP (Supplementary Information Figure S12C,D) and TAA (Supplementary Information Figure S13C,D). Consistent with that observed in LPS/D-GalN-induced ALI, ferrous iron accumulation and hepatocyte ferroptosis were observed in the liver (Supplementary Figures S12E–G and S13E–G). These data suggest that hepatic HIF-1α/TFR1 axis activation may be linked to the pathogenesis of drug- and toxin-induced ALI.

## 4. Discussion

In our initial experiments, we observed an increase in HIF-1α protein expression in the liver at different timepoints after the administration of LPS/D-GalN. HIF-1α has been implicated in hepatocyte death in liver diseases, such as APAP, sepsis, and fibrosis, which involve oxidative stress and inflammatory components [48,49]. The pathogenesis of ALI is characterized by massive cell death in the liver, a process that involves ferroptosis, apoptosis, necroptosis, pyroptosis, etc. [37,50]. Ferroptosis and pyroptosis were observed in the early and progressive phases, while apoptosis and necroptosis were significantly increased at 4 h post LPS/D-GalN treatment, which was consistent with previous findings [37,50]. Interestingly, our results revealed that HIF-1α is significantly correlated with ferroptosis and pyroptosis.

Ferroptosis is an iron-dependent form of cell death characterized by elevated lipid peroxidation, which is observed in various liver diseases [51]. Blocking ferroptosis can provide a cost-effective approach for preventing ALI [51]. To define the role of HIF-1α in hepatic ferroptosis under ALI induced by LPS/D-GalN, we generated HepHIF-1α^LSL/LSL^ mice and HIF-1α KO^Hep^ mice. Our study showed that HepHIF-1α^LSL/LSL^ promoted hepatocyte ferroptosis and liver injury in vivo, whereas HIF-1α KO^Hep^ exerted opposite effects in ALI induced by LPS/D-GalN. In vitro, two distinct classical ferroptosis inducers (RSL3 and ferric citrate) markedly triggered ferroptosis and cell injury in primary hepatocytes, which were alleviated by HIF-1α knockout and exacerbated by HIF-1α overexpression. Thus, our study elucidated the positive regulation of hepatic HIF-1α in ferroptosis under ALI condition and the pathological role in ALI.

Intracellular iron is indispensable for lipid peroxidation during ferroptosis [52]. The liver is the principal organ for iron storage and regulation [53,54], and dysregulation of the liver to maintain iron homeostasis has been reported in patients and animals with liver failure [16,17,55]. In mammals, transferrin receptor 1 (TFR1), is a vital endogenous regulator of iron homeostasis that imports iron into cells through transferrin-bound iron [15,17,52]. The upregulation of TFR1 expression has been shown to activate ferroptosis, and TFR1 inhibition or knockdown has been shown to efficiently block ferroptosis [19]. TFR1 has been identified as a target gene of HIF-1α [56,57]; however, the function of the HIF-1α/TFR1 axis in hepatocyte ferroptosis is not fully understood. In this study, RNA sequencing and Western blot analyses of livers from LPS/D-GalN-treated HepHIF-1α^LSL/LSL^ mice revealed that HIF-1α mediated iron levels through increasing TFR1 expression, promoting ferroptosis in hepatocytes. Furthermore, the chemical suppression of TFR1 efficiently reversed the increase in ferroptosis induced by HIF-1α in vitro and in vivo, suggesting TFR1 functions downstream of HIF-1α. More vitally, our results revealed that the HIF-1α/TFR1/ferroptosis axis is activated in the livers of mice injected with APAP and TAA [46] and in patients with ACLF. Therefore, hepatic HIF-1α regulates hepatic iron uptake through TFR1, leading to iron accumulation in liver, thereby mediating hepatocyte ferroptosis and ALI.

Recent studies argue that the effects of HIF-1α on ferroptosis are based on pathology and cell type specificity. On the one hand, HIF-1α alleviates ferroptosis in solid tumors, promoting drug resistance via SLC1A1 or the ARNTL/EGLN2/HIF-1α axis [29,30], while it protects against ferroptosis in hepatic stellate cells through SLC7A11, thereby mediating liver injury [28]. In addition, recent studies indicate HIF-1 protection against acute kidney injury via mitochondrial oxidative capacity, redox homeostasis, and autophagy [58]. On the other hand, other studies argue that HIF-1α promotes tissue damage in hippocampal neurons [26], testes [25], and diabetic renal injury [23,27] by activating HO-1. In our study, we demonstrated that hepatic HIF-1α facilitates ferroptosis via TFR1 upregulation and iron accumulation. Given the critical role of iron accumulation in ferroptotic lipid peroxidation, we speculate that this iron dysregulation mediates the pathological role of HIF-1α in acute liver injury.

Macrophage HIF-1α also plays a very important role in ALI. Our previous study found a new class of potent HIF-1α inhibitors (Cpd-4), which protect ALI via suppressing the production of IL-1β in macrophage [45]. While recent studies have indicated that HIF-1α in hepatocytes exacerbates ferroptosis in ALI induced by different etiology, dual inhibition of HIF-1α in both hepatocytes and macrophages may be a viable strategy for ALI. Thus, Cpd-4 was used to evaluate the effect on hepatocyte ferroptosis in vitro and in vivo. Consistent with hepatic HIF-1α knockout protecting against ferroptosis, Cpd-4 significantly inhibited ferroptosis triggered by classical ferroptosis inducers (RSL3 and FC) in primary hepatocytes. Notably, Cpd-4 effectively reduced ferroptosis in hepatocytes from HIF-1α^fl/fl^ mice, but not in hepatocytes from HIF-1α KO^Hep^ mice, indicating that Cpd-4 targets HIF-1α to inhibit ferroptosis. In vivo, we also revealed that Cpd-4 significantly reduced liver iron accumulation, ferroptosis, inflammation, and ultimately alleviated LPS/D-GalN-induced ALI.

ALI presents diverse therapeutic avenues under clinical investigation to target etiology, inflammation, and hepatocyte death [59]. Combining or using single medications that address multiple aspects of a condition can indeed yield superior outcomes. To date, a significant number of inhibitors targeting inflammation of macrophage and ferroptosis have shown therapeutic potential in preclinical studies, but some of them have many problems, such as poor absorption, side effects, and others [60]. HIF-1α could emerge as a promising candidate with anti-inflammation and anti-ferroptosis activities in ALI [12,45,61,62]. Although numerous HIF-1α inhibitors have been in clinical trials [63], none of them were developed to treat ALI. Our data indicated that HIF-1α may serve as a promising intervention target for ALI management.

## 5. Conclusions

In conclusion, our data indicated that the hepatic HIF-1α/TFR1 axis plays a vital role in triggering iron-mediated ferroptosis in ALI. HIF-1α activation increases iron accumulation and ferroptosis in hepatocytes. The pharmacological inhibition of HIF-1α in hepatocytes and macrophages is practical for ALI treatment.

## Data Availability

The original contributions presented in this study are included in the article/Supplementary Material. The original RNA Sequencing data presented in the study are openly available in GEO at GSE315731. Further inquiries can be directed to the corresponding author(s). Further inquiries can be directed to the corresponding author(s).

## Supplementary Materials

The following supporting information can be downloaded at: https://www.mdpi.com/article/10.3390/antiox15010081/s1, Figure S1: Increased HIF-1α expression is correlated with ferroptosis in the livers of mice with LPS/D-GalN-induced ALI.; Figure S2: Hepatocyte-specific HIF-1α overexpression exaggerates ferroptosis in vivo and in vitro. Figure S3: Hepatic HIF-1α regulates TFR1 expression and iron metabolism in ALI. Figure S4: Hepatic HIF-1α regulates iron homeostasis and ferroptosis. Figure S5: Hepatic HIF-1α regulates TFR1 expression and iron metabolism in ALI. Figure S6: Hepatic HIF-1α regulates iron homeostasis via TFR1 to promote ferroptosis. Figure S7: Hepatic HIF-1α regulates iron homeostasis via TFR1 to promote ferroptosis. Figure S8: The TFR1 inhibitor Ferristatin II alleviates LPS/D-GalN-induced ALI. Figure S9: Hepatocyte-specific HIF-1α ablation mitigates ferroptosis and LPS/D-GalN-induced ALI. Figure S10: The HIF-1α inhibitor Compound 4 alleviates hepatocyte ferroptosis in a HIF-1α dependent manner. Figure S11: The HIF-1α inhibitor Compound 4 alleviates LPS/D-GalN-induced ALI. Figure S12: Hepatic HIF-1α/TFR1 axis activation in APAP-induced ALI. Figure S13: Hepatic HIF-1α/TFR1 axis activation in TAA-induced ALI. Table S1: Reagents and chemicals used in this study. Table S2: Primary antibodies used in this study. Table S3: Sequences of primers used in this study. Table S4: The patient information of ACLF. References [64,65] are cited in the supplementary materials.
