# Supplementary material for: Hepatic Hypoxia-Inducible Factor 1α Mediates Ferroptosis via Transferrin Receptor 1 in Acute Liver Injury
Source: Antioxidants (Basel). 2026 Jan 8;15(1):81. doi: 10.3390/antiox15010081 (PMC12837155; doi:10.3390/antiox15010081)
Supplement: Supplementary file 1 [file antioxidants-15-00081-s001.zip › antioxidants-4026917-supplementary.pdf]

Supplementary Material for

Hepatic hypoxia-inducible factor 1α mediates ferroptosis via transferrin receptor 1 in acute liver injury

This word file includes:

Supplementary Methods

Supplementary Tables

Supplementary Methods

Antibodies and reagents

Antibodies and reagents are provided in Supplementary table 1-2.

Animal

Hepatocyte-specific HIF-1α knockout (HIF-1α-KO^{hep}) mice (C57BL/6J background) were generated by crossing the floxed HIF-1α mice (stock No: 007561, HIF-1α flox, Jackson Laboratory, Bar Harbor, Maine, USA) with Alb-Cre recombinase transgenic mice, the WT littermates (HIF-1α $^{fl/fl}$) were used as the control.

HepHIF-1α$^{LSL/LSL}$ mice (C57BL/6 background) were generated by cross-breeding ALB-Cre mice, which express Cre recombinase in hepatocytes under the control of the ALB promoter, with HIFdPA$^{fl/fl}$ mice, which lack proline hydroxylation sites for HIF degradation, the WT littermates (HIFdPA$^{fl/fl}$, HIF-1α$^{+/+}$) were used as the control. C57BL/6J mice (male, 7-8-week-old) were purchased from Beijing HFK Bio-Technology Co., Ltd.

Animal Model and approval

All animal experiments were conducted by a protocol approved by the Institutional Animal Care and Use Committee of Shanghai Institute of Materia Medica, Chinese Academy of Science. Adult male mice were used at ages as indicated for the respective experiments. ALI was induced by LPS/D-GalN in mice according to published research[1,5]. The mice were intraperitoneally (i.p.) injected with 100 μg/kg body weight LPS (Escherichia coli, O111:B4; Sigma-Aldrich, St. Louis, USA) and 700 mg/kg body weight D-GalN(D-galactosamine) (G0500; Sigma-Aldrich, St. Louis, USA) for indicated time.

For the study of LPS/ D-GalN -treated timepoints, mice were injected with LPS (100 μg/kg) and D-

GalN (700 mg/kg) (n=7 per group) or vehicle solution (n=7) for timepoints of 1-5 h.

For the study of HIF-1α overexpression mice (HepHIF-1α$^{LSL/LSL}$ mice)，the mice were divided into four groups: HIF-1α$^{+/+}$ treated with vehicle solution (n=8), HepHIF-1α$^{LSL/LSL}$ treated with vehicle solution (n=8), HIF-1α$^{+/+}$ treated with LPS/ D-GalN (n=11), HepHIF-1α$^{LSL/LSL}$ treated with LPS/ D-GalN (n=11). The LPS/D-GalN groups were injected with LPS (100 μg/kg) and D-GalN (700 mg/kg) for 4 h.

For the study of hepatic HIF-1α knockout mice (HIF-1α-KO^{hep}), the mice were divided into four groups: HIF1α$^{fl/fl}$ treated with vehicle solution (n=5), HIF-1α-KO^{hep} treat with vehicle solution (n=5), HIF1α$^{fl/fl}$ treated with LPS/D-GalN (n=12), HIF-1α-KO^{hep} treat with LPS/D-GalN (n=11). The LPS/D-GalN groups were injected with LPS (100 μg/kg) and D-GalN (700 mg/kg) for 4 h.

For the study of FerII treatment, the mice were divided into five groups: normal control group (n=10), vehicle solution treatment group (n=9), 10 mg/kg FerII treatment group (n=10), 30 mg/kg FerII treatment group (n=9), 10 mg/kg Lip-1 treatment group (n=9). FerII, Lip1 or vehicle solution were intraperitoneally injected three times in mice before LPS/D-GalN injection. The LPS/D-GalN groups were injected with LPS (100 μg/kg) and D-GalN (700 mg/kg) for 5 h.

For the study of Cpd-4 treatment, the mice were divided into four groups: normal control group (n=10), vehicle solution treatment group (n=9), 3 mg/kg Cpd-4 treatment group (n=9), 30 mg/kg Cpd-4 treatment group (n=9). Cpd-4 or vehicle solution were orally administrated three times before LPS/D-GalN injection. The LPS/D-GalN groups were injected with LPS (100 μg/kg) and D-GalN (700 mg/kg) for 5 h.

ALI was induced by APAP in mice according to published research[13]. The mice were starved for 12h and then intraperitoneally injected with 300 mg/kg APAP (Abs44055999, Absin) (n=6) or

vehicle solution (n=5) for 6 h.

ALI was induced by TAA in mice according to published research[31]. The mice were intraperitoneally (i.p.) injected with 100 mg/kg TAA (Abs42028342，Absin) (n=5) or vehicle solution (n=5) for 24 h.

Liver injury and Histological analyses

Plasma lactate dehydrogenase (LDH), alanine aminotransferase (ALT) and aspartate aminotransferase (AST) levels were measured by a JCA-BM6010/C Automatic Analyzer (JEOL, Tokyo, Japan) according to the manufacturer's instructions.

Histological pathology was performed as a standard protocol[32]. Briefly, liver tissues were collected and routinely embedded into paraffin. Liver sections were stained with hematoxylin staining (Ribiology, Shanghai, China; Yangming Medical Laboratory, Ningbo, China). The iron distribution in tissues was measured by 3,3'-Diaminobenzidine (DAB)-enhanced Perls' Staining [22,33](Yangming Medical Laboratory, Ningbo, China). The histological features of the tissues were observed under Brightfield and Fluorescence Slide Scanning System (Shenzhen Shengqiang Technology Co, Ltd., Shenzhen, China) and imaged.

Immunohistofluorescence

The protocol of immunohistofluorescence analysis followed that of our previous study [64,65] . After staining, the sections were scanned by Brightfield and Fluorescence Slide Scanning System (Shenzhen Shengqiang Technology Co, Ltd., Shenzhen, China). For quantification, 8-10 fields were randomly selected and calculated by ImageJ (NIH). All the data of immunohistofluorescence were relative to the number of nuclei.

RNA sequencing

RNA was isolated from the liver using TRIzol reagent. RNA sample quality control, cDNA library preparation, RNA sequencing, and bioinformatics analysis were performed by APExBIO Technology LLC (Shanghai, China).

Isolation and culture of primary hepatocytes

Primary hepatocytes were isolated from male C57BL/6J mice at 6-8 weeks of age. The mice were anesthetized and perfused with perfusion buffer and collagenase-I (0.48 mg/mL, LS004196, Worthington, Lakewood) through the portal vein at 37 °C. The liver of each mouse was cut, dispersed, filtered through a 70 mm cell strainer (Thermo Fisher Scientific) and spun at 700 r/min for 5 min at 4 °C. The cells were then resuspended in Hepato ZYME-SFM (17705021, GIBCO, Grand Island, NY) medium, and plated at the indicated density in a culture plate [32].

Primary hepatocytes were seeded in 96-well plates at the density of $2*10^4$ for 6 h in a mixture of low-glucose and adherent culture medium (4:1) supplemented with 10% FBS, 1x P/S,1x Glutathione. Primary hepatocytes were then cultured overnight in William's E medium containing 5% FBS, 1x P/S and 10 mM HEPES prior to any treatments[18].

Propidium iodide (PI) staining

Staining of live cells with PI was according to the manufacturer's instructions (Beyotime). Briefly, treated hepatocytes were incubated for 15 min with PI and DAPI in the culture medium. Cells were washed with PBS, followed by fluorescent imaging by Opera Phenix (PerkinElmer, Waltham, MA, USA). The numbers of PI^+ cells and total numbers of cells per field were counted, from which the percentage of PI^+ cell death was calculated.

Detection of iron, ROS, lipid peroxidation

Intracellular iron, ROS, and lipid peroxidation were quantified using FerroOrange (F374, Dojindo Laboratories), Dihydroethidium (DHE) (HYD0079, MedChemExpress), Liperfluo (L248, Dojindo Laboratories). Briefly, washed the cells with PBS, incubated with 1 μM FerroOrange, 10 μM DHE and 1μM Liperfluo for 30 min in a humidified and dark chamber at 37 °C. Fluorescent images were captured by Opera Phenix under the same conditions. Image J software was used for relative intensity analysis.

Western blot analysis

The protocol of Western blot analysis followed that of our previous study[65]. Briefly, cells or liver tissues were lysed with RIPA buffer containing 1 μM PMSF (Beyotime). Protein concentration was determined with BCA reagent from Beyotime. Proteins (25 μg) were separated on SDS-polyacrylamide gels. After being transferred onto NC membranes, blots were blocked with 5% fat-free milk and probed subsequently with primary antibodies and horseradish peroxidase-conjugated secondary antibodies. Antigens were visualized with an enhanced chemiluminescence kit (Epizyme, China). β-actin was used to monitor protein loading and transferring. For quantification, the protein bands were analyzed with ImageJ software (NIH, USA).

Quantitative RT-PCR analysis

Total RNA was isolated using TRIzol reagent (15596018, Life Technologies). Then, RNA (1 μg) was reverse transcribed to cDNAs using PrimeScript Reverse Transcriptase (Takara). The resulting cDNAs were amplified using 2×SYBR Green qPCR Master Mix (B21702, Bimake) and a Stratagene Mx3005P instrument (Agilent Technologies). Expression was normalized to housekeeping gene (18S rRNA). The relative quantity of mRNA was normalized to the control

group using the ΔΔCT method. The Primer sequence details are shown in Supplementary Table 3.

Statistical analysis

Statistical analyses were used GraphPad Prism 8.0 and presented as mean ±SEM. Student's t test was performed to compare the differences between two groups. Comparisons between the three or more groups were analyzed using one-way ANOVA/ Fisher's LSD test (more than two groups). $p < 0.05$ was considered significantly different.

Supplementary Tables

Table S1 Reagents and chemicals used in this study.

Reagents and chemicals	Catalogue	Company
Collagenase Type1	LS004196	Worthington
Ferric citrate	XW35225071	Sinopharm Chemical Reagent Co., Ltd
Lipopolysaccharides(LPS)	L2630	Sigma
D-galactosamine (D-GalN)	G0500	Sigma
Ferristatin II	HY-16214	MedChemExpress
Liproxstatin-1	BD631059	Bidepharm
Liperfluo	L248	DOJINDO
FerroOrange	F374	DOJINDO
Dihydroethidium	HY-D0079	MedChemExpress
PI	C1075M	Beyotime
RSL3	HY-100218A	MedChemExpress

FBS	10099141	Gibco
Williams' E medium	W4128	Sigma
DMEM low Glucose Pyrurate	11885084	Gibco
HepatoZYME-SFM	17705021	Gibco
Hepes	60117ES60	Yeasen
Percoll	P1644	Sigma
GlutaMAX	35050061	Gibco
Aspartate Aminotransferase Assay Kit	KH1001-2	KINGSBIO
Alanine Aminotransferase Assay Kit		
Lactate Dehydrogenase Assay Kit	KH1002-2	KINGSBIO
Acetaminophen (APAP)	KH1029-3	KINGSBIO
Thioacetamide(TAA)	Abs44055999	Absin
Tris Base	Abs42028342	Absin
Trincine	60102ES76	Yeasen
N,N-Methylenebis	S16030	Yuanye
	V900301	Sigma

Table S2 Primary antibodies used in this study.

Antibodies	Host	Dilution	Catalogue	Company
**Western blotting**				
HIF-1α	Rabbit pAb	1:1000	GTX127309	GeneTex
TFR1	Mouse mAb	1:1000	AB269513	Abcam
MLKL	Rabbit pAb	1:1000	AP14272B-ev	Abcepta
PTGS2	Rabbit mAb	1:1000	12282S	Cell Signaling Technology
HO-1	Rabbit mAb	1:1000	43966S	Cell Signaling Technology
NLRP3	Rabbit mAb	1:1000	15101S	Cell Signaling Technology
c-Caspase3	Rabbit mAb	1:1000	9664L	Cell Signaling Technology
α-Tubulin	Rabbit pAb	1:5000	2144S	Cell Signaling Technology
**Immunofluorescence**				
HIF-1α	Mouse pAb	1:50	NB100-105	NOVUS
PTGS2	Rabbit pAb	1:500	AB15191	Abcam
4-HNE	Mouse pAb	1:300	AB48506	Abcam
TFR1	Rabbit pAb	1:100	AF8136	Beyotime
MPO	Goat pAb	1:200	AF3667	R&D systems

Table S3 Sequences of primers used in this study.

Species	Gene		Primer
**Real-time PCR**			
Mouse	Tnf-α	Forward	GAAGTTCCCAAATGGCCTCC

	Gene ID: 21926	Reverse	TTGTCACTCGAATTTTGAGAAGATG
Mouse	Il-1β	Forward	TGGACCTTCCAGGATGAGGACA
	Gene ID: 16176	Reverse	GTTCATCTCGGAGCCTGTAGTG
Mouse	β-actin	Forward	CATTGCTGACAGGATGCAGAAGG
	Gene ID: 11461	Reverse	TGCTGGAAGGTGGACAGTGAGG
Mouse	Ptgs2	Forward	CTGCGCCTTTTCAAGGATGG
	Gene ID: 19225	Reverse	GGGGATACACCTCTCCACCA
Mouse	Tfrc	Forward	ACCATTGTCATATACCCGGTTCA
	Gene ID: 22042	Reverse	CAATAGCCCAAGTAGCCAATCAT
Mouse	Hif-1α	Forward	TGCTCATCAGTTGCCACTT
	Gene ID: 15251	Reverse	GTTGGGGCAGTACTGGAAAG
Mouse	Il-6	Forward	TACCACTTCACAAGTCGGAGGC
	Gene ID: 16193	Reverse	CTGCAAGTGCATCATCGTTGTTC
Mouse	Mcp-1	Forward	GCTACAAGAGGATCACCAGCAG
	Gene ID: 20296	Reverse	GTCTGGACCCATTCCTTCTTGG
Mouse	18S rRNA	Forward	GTAACCCGTTGAACCCCATT
	Gene ID: 19791	Reverse	CCATCCAATCGGTAGTAGCG
**Genotyping**			
Mouse	ALB-cre	Forward	TGCTGTTTCACTGGTTATGCGG
		Reverse	TTGCCCCTGTTTCACTATCCAG
Mouse	Hif-1α-flox	Forward	TGCTCATCAGTTGCCACT T
		Reverse	GTTGGGGCAGTACTGGAA AG

Mouse	Hif-1α-dpa	Forward	CGTGATCTGCAACTCCAGTC
		Reverse	CCGGTAGAATTCCTGCAGGTCGAGGG
		Reverse	GGAGCGGGAGAAATGGATATG

Table S4 The patient information of ACLF.

Type	Sex	Age	AST(U/L)	ALT(U/L）	INR	TBIL(uM)
Normal control	Female	43	13	10	1.06	3.8
Normal control	Female	33	31	61	0.99	2
Normal control	Female	29	18	32	1.03	3.4
Normal control	Male	30	22	39	1.07	7.5
Normal control	Female	32	14	13	1.02	4.3
Normal control	Female	26	18	19	1.01	2.4
ACLF	Male	40	97	129	3.01	547.1
ACLF	Male	53	94	83	2.19	503.3
ACLF	Female	67	123	109	2.41	513.9
ACLF	Female	53	214	114	1.89	366.8
ACLF	Male	52	2608	1341	1.94	48
ACLF	Male	48	1858	508.1	1.41	116.2
ACLF	Male	44	163	92	2.38	814.1
